# Impact of laparoscopic repair on type III/IV giant paraesophageal hernias: a single-center experience

**DOI:** 10.1007/s10029-023-02851-7

**Published:** 2023-08-29

**Authors:** E.-S. A. El-Magd, A. Elgeidie, Y. Elmahdy, M. El Sorogy, M. A. Elyamany, I. L. Abulazm, A. Abbas

**Affiliations:** 1 Faculty of Medicine, Mansoura University, Mansoura, Egypt; 2 Department of General Surgery, Faculty of Medicine, Gastrointestinal Surgical Center GISC, Mansoura University, Gehan Street, Mansoura, 35511 Al Dakahlia Governorate Egypt

**Keywords:** Giant paraesophageal hernia, Tailored surgical procedure, Recurrence, Risk factors, GIQLI score

## Abstract

**Purpose:**

Giant paraesophageal hernia (GPEH) is a challenging problem for surgeons because of its high recurrence rate. This study was conducted to compare the outcomes in type IV vs. type III GPEHs after laparoscopic repair. Other outcomes included peri-operative morbidity and long-term quality of life.

**Methods:**

A retrospective analysis of 130 GPEH patients in a period between 2010 and 2019 underwent a tailored laparoscopic repair in our tertiary center with a minimum follow-up of 48 months. Operative steps included hernial sac excision, crural repair, relaxing incisions, and mesh cruroplasty with special indications.

**Results:**

The study enrolled 90 patients with type III and 40 patients with type IV GPEH. Type IV GPEH patients were older, more fragile, and scored worse on ASA classification, aside from having a more challenging surgical technique (wider crura, weaker muscles, increased need for release incisions, and mesh cruroplasty).Type IV GPEHs had a prolonged operative durations, and a higher conversion rate. Additionally, the same group showed increased morbidity, mortality, and re-operation rates. With a mean follow-up of 65 months (range 48–150 months), the incidence of recurrence was 20.7%, with an increased incidence in type IV GPEH (37.5% vs. 13.33% in type III GPEH). Type IV GPEH, low pre-operative albumin, larger crural defect, and low surgeon experience were significant risk factors for recurrence after laparoscopic repair of GPEH.

**Conclusion:**

Type IV GPEH has a higher peri-operative morbidity and recurrence rate; so, a more tailored laparoscopic repair with a high surgeon experience is needed.

## Introduction

Hiatus hernia (HH) is a common disease, especially in older adults, with a rising incidence over the last two decades [1]. It is the most common form of diaphragmatic hernia, and it is generally classified into four categories: type I (sliding), type II (pure paraesophageal), type III (mixed), and type IV (complex) [2].

Giant paraesophageal hernia (GPEH), including types III and IV, is characterized by herniation of more than one-third of the stomach into thoracic cavity and accounts for 5–10% of all hiatus hernias[3]. The clinical presentation of GPEH varies from instantly asymptomatic to intermittent mechanical symptoms, such as pain, dysphagia, or dyspnea [4].

Surgical treatment of GPEH is highly recommended by the Society of American Gastrointestinal and Endoscopic Surgeons (SAGES) because they are prone to life-threating complications like gastric volvulus and strangulation [5].

Recurrence rates after surgical treatment of GPEH are relatively high and varies from 15 to 66% according to definition of recurrence. Surgeons have to deal with several challenges to achieve the best outcomes and decrease that high recurrence rate [6, 7].

The concept of radial tension, caused by a very wide hiatus, and axial tension, caused by an apparent short esophagus, is widely accepted among authors as the main incriminating factors in the process of recurrence after surgical repair [8].

Several techniques have been developed to overcome these technical obstacles. The ideal method to address the presence of radial tension is widely variable, and the utilization of a crural release incision or the use of mesh cruroplasty has been advocated. However, the durability has been questioned. Also, several complications of mesh cruroplasty have been described [3, 9, 10].

The incidence of short esophagus is still a matter of debate; some authors believe that such an entity does not exist and adequate mediastinal mobilization always achieves the desired intra-abdominal esophageal length [11, 12].Contrarily, other studies reported an incidence of short esophagus reaching up to 33%, and proposed an additional procedures to overcome this matter, e.g., intentional vagotomy or Collis gastroplasty [13].

GPEH, therefore, is considered a challenging operation, and although many authors have studied the outcome after GPEH repair, the ideal approach is still a matter of surgical debate. In this paper, we compare the outcomes in type IV vs. type III GPEHs after laparoscopic repair. Other outcomes included peri-operative morbidity and long-term quality of life.

## Patients and methods

This retrospective study was conducted on 130 patients who underwent laparoscopic repair of GPEH during the period between January 2010 and January 2019. After gaining approval from our local scientific committee (IRB code: R.22.04.1695), the data of these patients were collected from our database and then reviewed.

The diagnosis of GPEH was defined as > 30% of prolapsed stomach with or without other organs prolapsed into the chest on pre-operative imaging [14, 15]. Type I, type II, and recurrent hernia cases were excluded from our analysis.

Pre-operative preparation included proper history taking, gastrointestinal quality-of-life index (GIQLI score questionnaire) [16], physical examination, and laboratory work-up. The diagnosis of hiatus hernia was made by upper GI endoscopy, barium esophagogram, and computed tomography (CT) scanning.

The barium study was done for objective evaluation (Reviewer #2) of the hernia and to calculate the hernial length by measuring the distance between the diaphragm and the upper level of the herniated stomach (Reviewer #3). CT scanning was done for identification of other hernia contents beside the stomach. Additionally, all patients were assessed for endoscopic reflux and classified according to the Los Angeles classification [17].

All patients were assessed by the anesthetic team and classified according to American Society of Anesthesiologists (ASA) score [18], and all procedures were performed under general anesthesia.

### Tailored laparoscopic repair procedure

The laparoscopic procedure was performed via the classic five-port design (1 camera, 2 working, and 2 assistant ports).

### Hernial sac excision

**Fig. 1:**
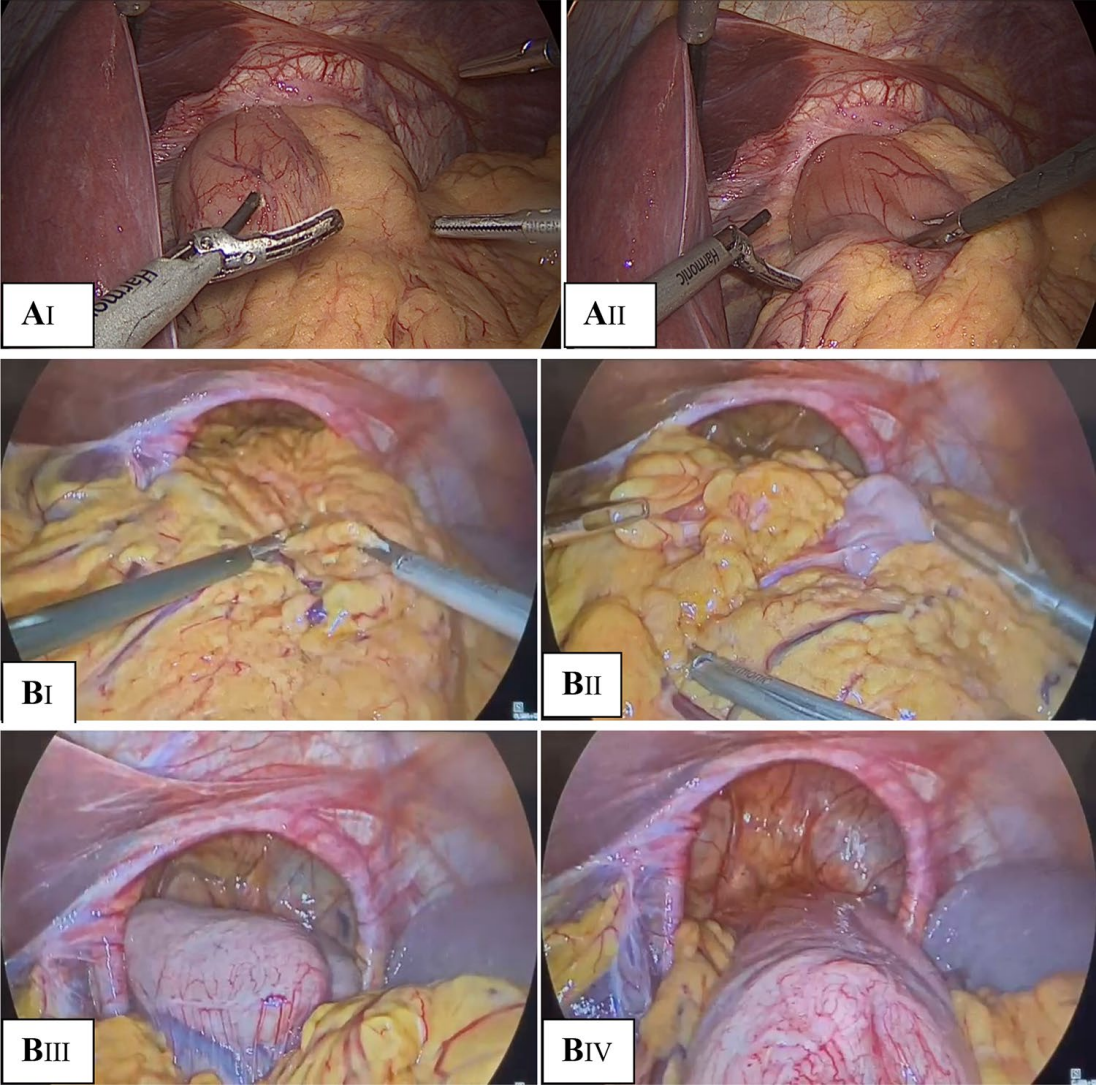
**A** Type III GPEH containing about half of the stomach (AI and AII: reduction of the stomach). **B** Type IV GPEH containing the omentum, transverse colon and the whole stomach with secondary volvulus (BI and BII: reduction of the omentum and colon. BIII and BIV: reduction of the whole stomach

After reduction of the GPEHs content as shown in Fig. 1, the hernial sac was dissected from the mediastinum and reduced to the abdominal cavity, and the width of the hiatus was measured and recorded.

**Fig. 2:**
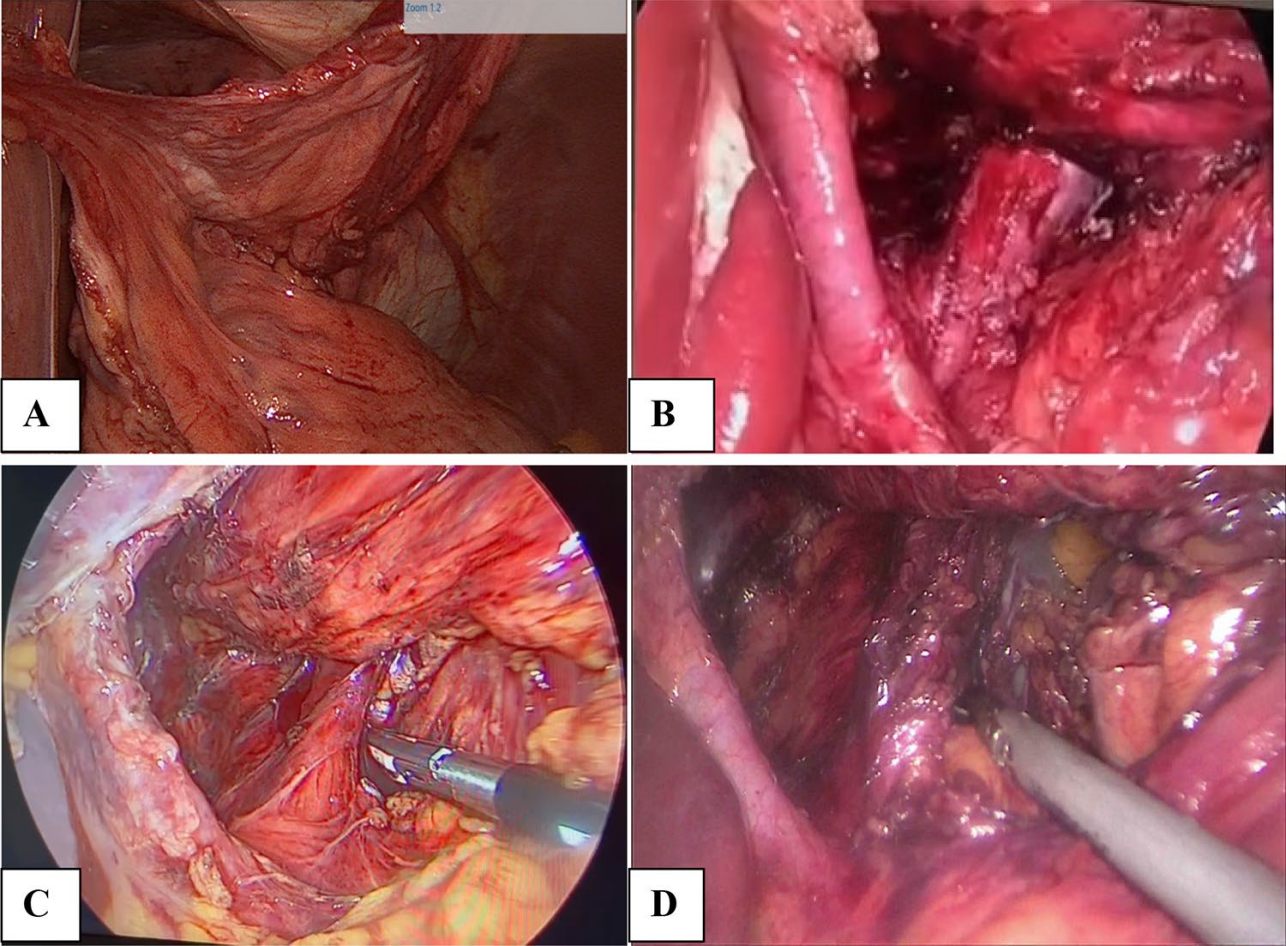
**A** Hernia sac excision, **B** type III GPEH with hiatal dissection. **C** and **D** Type IV GPEH with hiatal dissection

The defect size was measured with the aid of the non-traumatic laparoscopic grasper, considering the distance between the two edges of that tool when it was opened, which was 2 cm. The hernial content was reduced to the abdominal cavity with careful adhesiolysis and excision of the hernial sac, as shown in Fig. 2.

### Crural repair

The method for crural closure was dependent upon the tension across the hiatus, integrity of the crural muscles, and their covering fascia. Crural radial tension was recognized simply when a tied slipped knot failed to keep both crural pillars in place.

Stitches were taken anteriorly or posteriorly to the esophagus. In some cases, we needed both anterior and posterior sutures.

The main determinant of the site of crural sutures was to avoid esophageal angulation. We usually started by posterior repair. However, when there was an apparent esophageal angulation, we replaced some of posterior sutures by anterior ones to decrease the risk of esophageal angulation and the risk of post-operative dysphagia (Reviewer #3).

The placement of sutures was guided by a 38-Fr bougie, ensuring its smooth passage through the diaphragmatic hiatus with no kink or resistance. The Bougie was used in both crural repair and fundoplication (Reviewer #3).

### Esophageal lengthening technique

**Fig. 3:**
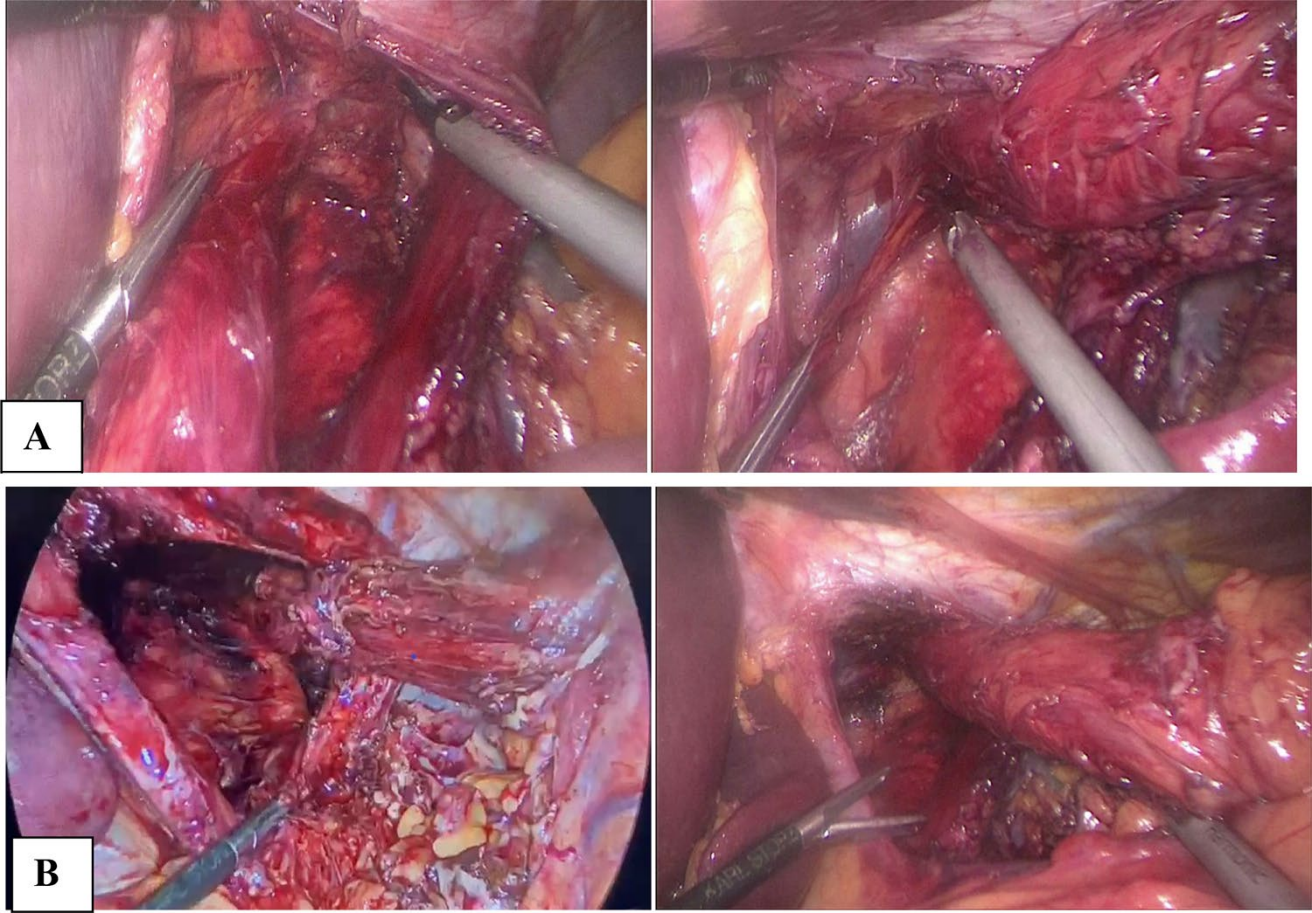
Extensive mediastinal dissection. **A** Antero-lateral and posterior mediastinal dissection. **B** 2.5–3 cm intra-abdominal esophagus with minimal traction

A short esophagus was defined as inability to get the gastroesophageal junction below the diaphragm, as published by Herbella et al. [19]. We performed extensive mediastinal mobilization in such patients till we achieved at least 2.5–3cm esophageal length below the diaphragm, with no need for a Collis gastroplasty, as shown in Fig. 3

### Relaxing crural incision

**Fig. 4:**
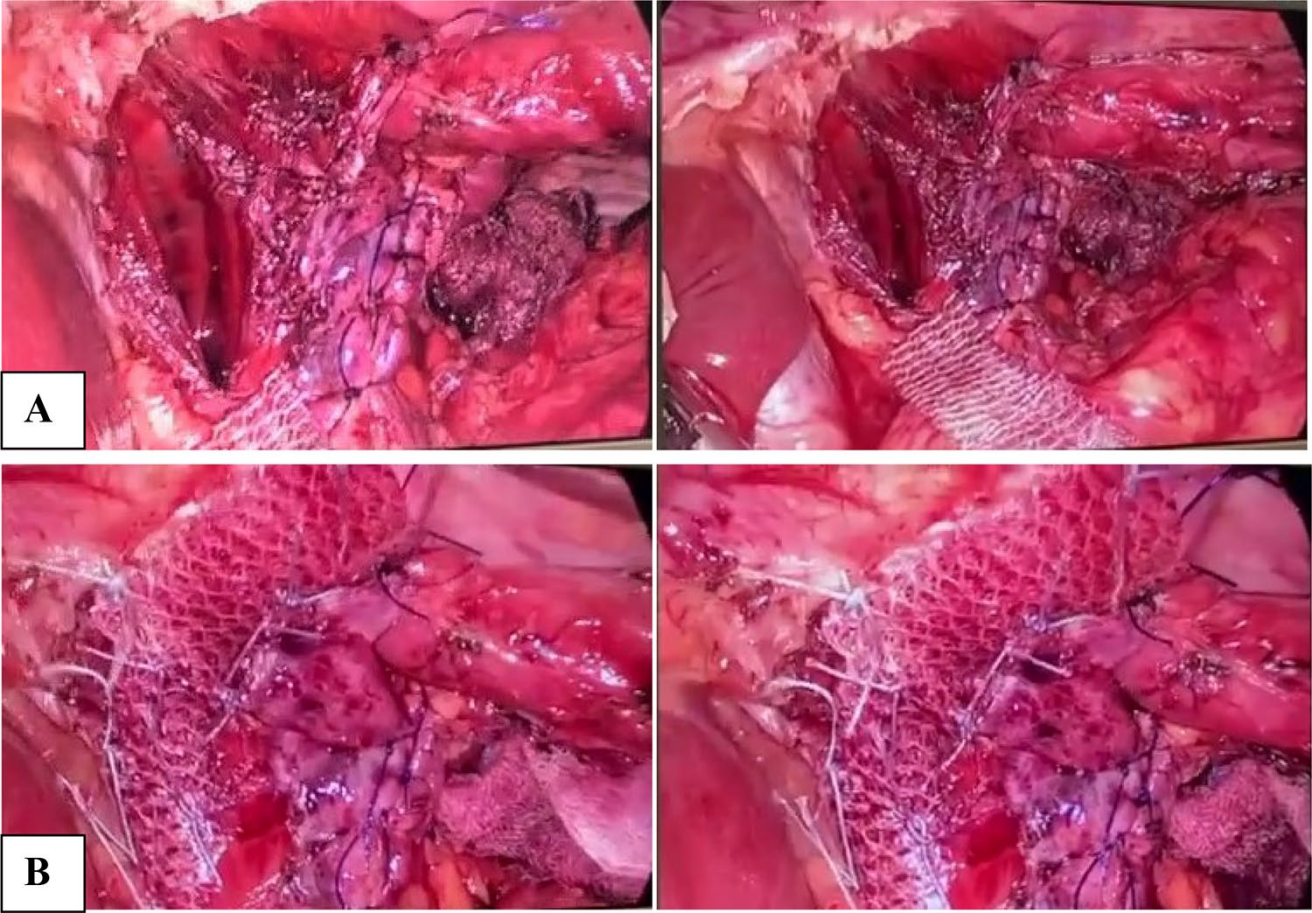
**A** Right-sided relaxing incision. **B** Longitudinal composite mesh over the relaxing incision

We performed either right or left crural relaxing incisions to allow tension-free crural closure. Only with weak crural muscles or/and with violated crural fascia, the site of the incision was reinforced by a rectangle-shaped composite mesh, overlying the crural incision site, as shown in Fig. 4.

### Mesh cruroplasty

Mesh cruroplasty was indicated either in combination with release incisions or when we encountered weak crural muscles with violated crural fascia.

The state of the crural muscles was determined subjectively. When we noticed that the muscles were easily torn or dissected with traction, it was taken as a mark for weakness (Reviewer #3).

**Fig. 5:**
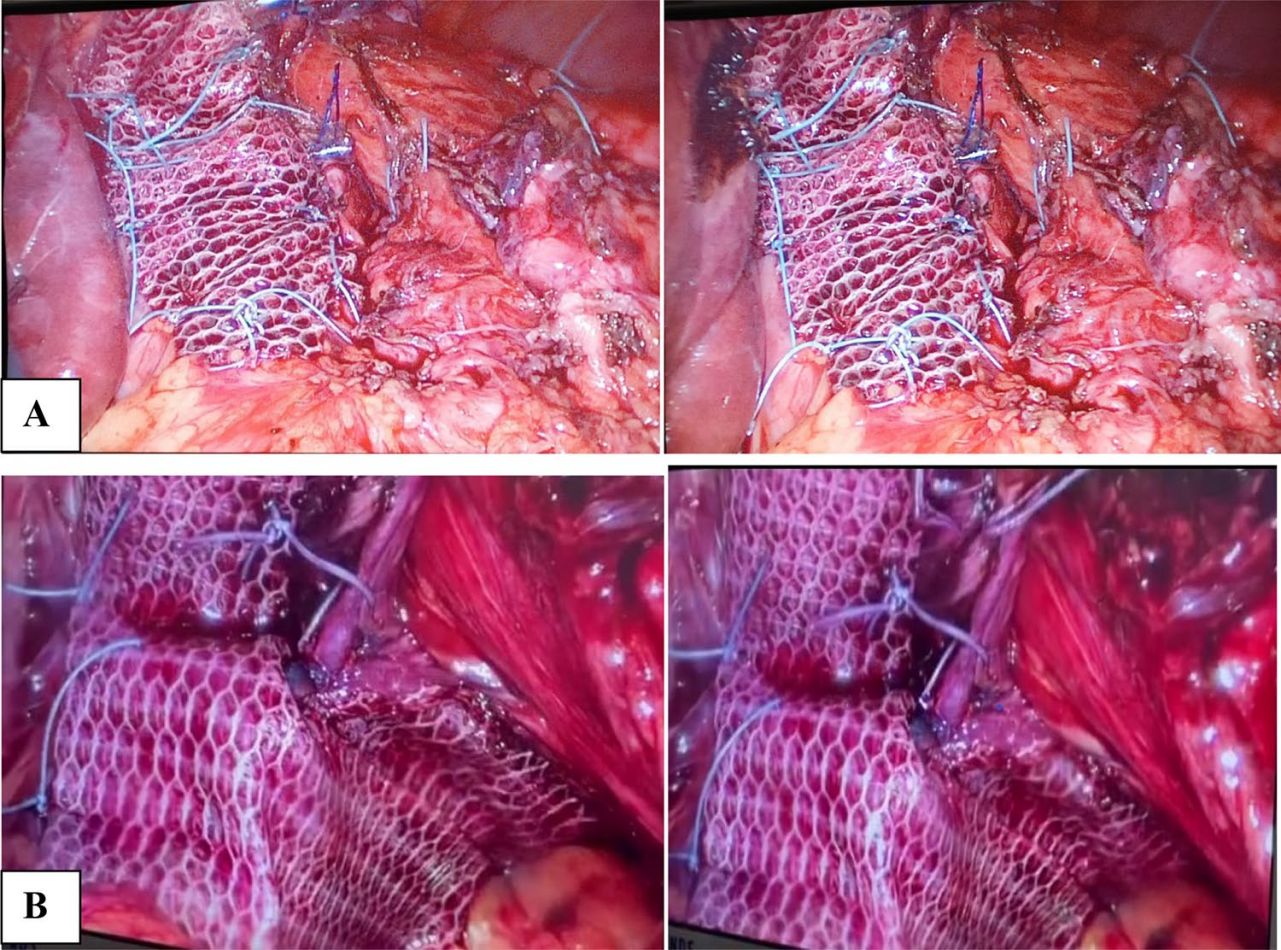
**A** Rectangle-shaped composite mesh over the relaxing incision. **B** U-shaped composite mesh over the crural repair

Regarding its configuration, either rectangle-shaped over the release incision, or U-shaped mesh over the hiatal repair as shown in Fig. 5.

Initially, we used synthetic polypropylene mesh (only in three patients) in U-shaped configuration (Reviewer #3), but due to mesh-related complications, we shifted to composite mesh, Parietex™ Composite (Covidien, New Haven, CT, USA) (Reviewer #3).

Noteworthy, biological mesh was not an option due to limited availability and a very high cost.

Two patients with Mesh erosion of the esophagus and stomach (with polypropylene mesh type). The first patient presented with severe dysphagia one month after the operation, and esophagogastroduodenoscopy revealed lower esophageal erosion by the mesh. The second patient presented with gastric fistula two weeks after the primary procedure, associated with peritonitis. Both patients were managed by proximal esophagogastrectomy with en bloc resection of the mesh (Reviewer #2).

### Gastropexy

Additional gastropexy was performed in all patients who had intra-operative volvulus, or when the operator found the stomach floppy and extremely mobile after its reduction to the abdominal cavity. The gastropexy was done by fixating the stomach to either transverse mesocolon, diaphragmatic crus, or abdominal wall.

We choose the site of gastropexy according to intra-operative findings. When there is a gastric volvulus, we preferred to choose the site of gastropexy to the transverse mesocolon (Posterior gastropexy). However, in some cases, the transverse colon was thin, or very rich in vasculature. In these cases, we choose the abdominal wall (Anterior gastropexy). When there is a wrap and the retro-esophageal space was relatively wide, we choose to fix of wrap to the crura (Fundopexy), to avoid rotation or twist of the wrap aiming to decrease the dysphagia symptoms (Reviewer #2) and (Reviewer #3).

### Fundoplication

Regarding the types of fundoplication performed, it is highlighted and already mentioned in Table 2. The choice of the fundoplication type (Dor, Toupet, or Nissen) was dependent on the creation of a floppy gastric valve (not dependent on manometric findings and that is one limitation of our research) (Reviewer #3).

### Post-operative care

After the operation, close monitoring was done for all patients, oral fluids were allowed in the 1st post-operative day, the patients were discharged home on the subsequent day, unless complications were encountered. Peri-operative complications were classified according to the Clavien–Dindo classification [20].

### Follow-up

Regular follow-up visits were scheduled for all patients, and we excluded patients who were dropped during the follow-up plan. Patients were interviewed for presence of any symptoms. We performed a follow-up upper GI endoscope and esophagogram at 1, 3, and 4 years after the procedure to evaluate post-operative recurrence. The GIQLI questionnaire was also repeated during the same previous visits. Clinical recurrence was defined in the presence of symptoms and documented anatomical failure by endoscopy and/or barium examination.

### Outcomes

Our main outcome was to compare the outcomes in type IV vs. type III GPEHs after a tailored surgical procedure. Other outcomes included peri-operative morbidity and long-term quality of life.

### Statistical analysis

The collected data were coded, processed, and analyzed using SPSS program (version 22) for MacOS. The Kolmogorov–Smirnov test was used to identify continuous variable distribution. Normally distributed variables were expressed as mean and standard deviation, while non-normally distributed variables were expressed as median and range. The Chi-square test was used to compare between categorical variables. Independent *t* test was used to compare normally distributed variables across groups, while Mann–Whitney test was used to compare non-normally distributed variables. Regression analysis was done to define the significant predictors for peri-operative morbidity and post-operative recurrence. A *p* value < 0.05 was considered significant.

## Results

### Baseline demographic and clinical characteristics

Out of the included 130 GPEH patients, 90 patients had type III GPEH, whereas the remaining 40 patients had type IV. The mean age of our study population was 55.8 years, and it was significantly higher in type IV GPEH (63.2 vs. 52.6 years in patients with type III GPEH—*p* < 0.001).

We included 67 males and 63 females, and the mean value of their BMI was 26.1 kg/m^2^. The chief presentation in both types of hernia was heartburn (48.5%), while dysphagia was the 2nd most common presentation in type IV GPEH (18 cases). Other complaints included regurgitation, epigastric pain, chest pain, vomiting, and easy fatiguability.

The prevalence of medical co-morbidities was comparable between type III and type IV GPEH patients, apart from cardiovascular disease that increased significantly in type IV GPEH patients (*p* < 0.001). In addition, patients with type IV GPEHs expressed higher ASA classes compared to the other group (*p* = 0.049).

Peptic stricture was found in five patients with type III GPEH on endoscopic examination (5.6%), while cameron ulcer was detected in 6.6% and 17.5% of patients with type III and IV GPEHs, respectively.

The length of the hernia was measured pre-operatively in the barium study, by estimating the distance between the top of gastric folds to the crural pinch. While percentage of the hernia size was estimated intra-operatively by the operating surgeon to the nearest 10%, depending on the incisura angularis as our main landmark (representing a midway along the stomach 50%) (Reviewer #3).

Even in gastric volvulus, we initially located the incisura angularis after gastric reduction. Then, we subjectively estimated the herniated gastric volume like before (Reviewer #3).

The pre-operative hernia length was significantly higher in type IV GPEH patients (*p* < 0.001). Moreover, the mean intra-operative percentage of herniated stomach was 58.2% and it was statistically higher in type IV GPEH patients (76.9% vs. 49.9% in type III patients). The previous data are summarized in Table 1.

**Table 1:** Demographic data of included patients

Variable	All patients (130)	Type III hernia (90)	Type IV hernia (40)	*p* value
Age (mean, SD)	55.8 (13.3)	52.6 (12.3)	63.2 (12.8)	< 0.001**
Sex				
Male	67 (51.5)	49 (54.4)	18 (45)	0.320*
Female	63 (48.5)	41 (45.6)	22 (55)	
BMI (mean, SD)	26.1 (3.7)	26.3 (3.6)	25.6 (4)	0.311**
Hgb (mean, SD)	10.8 (1.7)	11 (1.7)	10.5 (1.8)	0.177**
Albumin (mean, SD)	4 (0.3)	4 (0.2)	4 (0.3)	0.482**
Comorbidities				
DM	8 (6.2)	5 (5.6)	3 (7.5)	0.670*
Cardiovascular disease	33 (25.4)	15 (16.7)	18 (45)	< 0.001*
Chest disease	36 (27.7)	24 (26.67)	12 (30)	0.7
Smoking	36 (27.7)	26 (28.9)	10 (25)	0.647*
ASA				
I	67 (51.5)	50 (55.6)	17 (42.5)	0.049*
II	43 (33.1)	31 (34.4)	12 (30)	
III	20 (15.4)	9 (10)	11 (27.5)	
Clinical presentation				
Heart burn	63 (48.5%)	44 (48.8%)	19 (47.5%)	0.88*
Regurgitation	49 (37.7%)	33(36.7%)	16 (40%)	0.71*
Epigastric pain	56 (43.1)	41 (45.6)	15 (37.5)	0.392*
Chest pain	41 (31.5)	29 (32.2)	12 (30)	0.801*
Dysphagia	49 (37.7)	31 (34.4)	18 (45)	0.252*
Vomiting	20 (15.4)	11 (12.2)	9 (22.5)	0.134*
Fatigability	27 (20.8)	18 (20)	9 (22.5)	0.746*
Barret’s esophagus	4 (3.1)	3 (3.3)	1 (2.5)	0.8*
Endoscopy finding				
No GERD	2 (1.5)	1 (1.1)	1 (2.5)	0.068*
G A reflux	46 (35.4)	31 (34.4)	15 (37.5)	
G B reflux	56 (43.1)	43 (47.8)	13 (32.5)	
G C reflux	21 (16.2)	10 (11.1)	11 (27.5)	
Peptic stricture	5 (3.8)	5 (5.6)	–	
Cameron ulcer	13 (10)	6 (6.7)	7 (17.5)	0.061
Other hernia				
Inguinal	11 (84.6)	7 (77.8)	4 (100)	0.305*
Umbilical	2 (15.4)	2 (22.2)	–	
Hernia content				
Stomach–omentum	89 (68.5)	89 (98.9)	–	
Stomach–omentum–colon	39 (30)	1 (2.6)	38 (95)	< 0.001*
Stomach–omentum–spleen	1 (0.8)	–	1 (2.5)	
Stomach–spleen Pancreas	1 (0.8)	–	1 (2.5)	
Percentage of stomach herniated (mean, SD)	58.2 (21.9)	49.9 (16.7)	76.9 (20.7)	< 0.001**
Pre-operative hernia length (mean, SD)	9.9 (4.2)	8.2 (1.9)	13.9 (5.2)	< 0.001**

*Chi-square test **independent *t* test

### Operative data

Conversion to open surgery was needed in 6 cases (4.6%), with a significant increase in type IV GPEH, although surgeon experience was statistically comparable between the two groups (*p* = 0.166).

The indications for conversion were (massive bleeding from short gastric vessels (two patients), sizable pleural injury causing hemodynamic instability (two patients) and esophageal injury (two patients) (Reviewer #2).

No significant difference was found between both types of GPEH regarding the associated organ injuries or the presence of an apparent short esophagus.

However, type IV GPEH patients had significantly larger hiatal defect, weaker crural musculature, more bared diaphragmatic fascia, and higher incidence of gastric volvulus. Subsequently, they showed a significant increase in the need for release incision, mesh cruroplasty, and gastropexy procedures (*p* < 0.001).

Type IV GPEH patients also showed a significant increase in the number of sutures needed to close the hiatus, more prolonged operative time, and more intra-operative blood loss. The previous data are presented in Table 2.

**Table 2:** Intra-operative and post-operative data

Variable	All patients	Type III	Type IV	*p* value
		hernia	hernia	
Crura defect size	6.8 (1)	6.3 (0.7)	7.9 (0.9)	< 0.001**
Gastric volvulus no. (%)	37 (28.5)	11 (12.2)	26 (65)	< 0.001*
Additional gastropexy no. (%)	53 (40.8)	28 (31.1)	25 (62.5)	0.001*
Gastropexy method no. (%)				
Mesocolon	32 (60.4)	16 (57.1)	16 (64)	0.754*
Crus of diaphragm	15 (28.3)	8 (28.6)	7 (28)	
Abdominal wall	6 (11.3)	4 (14.3)	2 (8)	
Apparent Short esophagus	24 (18.5)	15 (16.7)	9 (22.5)	0.429*
Operator experience no				
< 10 cases per year	35 (26.9)	21 (23.3)	14 (35)	0.166*
≥ 10 cases per year	95 (73.1)	69 (76.7)	26 (65)	
Conversion	6 (4.6)	2 (2.2)	4 (10)	0.049*
Intra-operative injuries no				
Esophageal	2 (9.5)	–	2 (18.2)	0.283*
Pleural	13 (61.9)	6 (60)	7 (63.6)	
Short gastric	4 (19)	3 (30)	1 (9.1)	
Gastric wall	1 (4.8)	1 (10)	–	
Spleen	1 (4.8)	–	1 (9.1)	
Relaxing incision no. (%)	32 (24.6)	14 (15.6)	18 (45)	< 0.001*
Repair method no. (%)				
Primary suturing	114 (87.7)	85 (94.4)	29 (72.5)	< 0.001*
Mesh use	16 (12.3)	5 (5.6)	11 (27.5)	
Overlying crural fascia no.				
Preserved	119 (91.5)	87 (96.7)	32 (80)	0.002*
Bared	11 (8.5)	3 (3.3)	8 (20)	
Crura muscle condition no.				
Weak	28 (21.5)	13 (14.4)	15 (37.5)	0.003*
Good	102 (78.5)	77 (85.6)	25 (62.5)	
Wrap type no. (%)				
Nissen	101 (77.7)	72 (80)	29 (72.5)	0.638*
Dor	8 (6.2)	5 (5.6)	3 (7.5)	
Toupet	21 (16.2)	13 (14.4)	8 (20)	
Suture material no. (%)				
Silk	57 (43.8)	40 (44.4)	17 (42.5)	0.132*
Ethibond	41 (31.5)	32 (35.6)	9 (22.5)	
Prolene	32 (24.6)	18 (20)	14 (35)	
Operative time (mean, SD)	143.9 (50.8)	129.9 (27.3)	175.5 (73.1)	< 0.001**
Blood loss (mean, range)	180.9 (50–600)	158.7 (50–500)	231 (50–600)	0.003***
Total crura suture number (mean, SD)	4.5 (0.6)	4.3 (0.5)	4.9 (0.7)	0.004**
Posterior crura suture number (mean, SD)	3.8 (0.7)	3.7 (0.7)	4.1 (0.6)	< 0.001**
Anterior crura suture number (mean, range)	0.6 (0–2)	0.6 (0–2)	0.8 (0–2)	0.031***

*Chi-square test

**Independent *t* test

***Mann–Whitney test

### Peri-operative morbidity and mortality

Type IV GPEH patients had longer hospital stays and more serious complications according to the Cliven Dindo classification. Six patients of type IV GPEH needed re-operation (15%), and we had two patients (5%) with post-operative mortality (one patient due to sepsis of gastric fistula caused by mesh erosion and the other one due to sepsis from chest infection).

Major peri-operative morbidity (Clavien–Dindo > 3):

In Type III GPEH:

- Two patients with pneumonia
- One patient with hydro-pneumothorax

In Type IV GPEH:

- Two patients of acute respiratory distress due to acute herniation (re-operation)
- Two patients of mediastinal hematoma (re-operation)
- Two patients with mesh erosion of the esophagus and stomach (with polypropylene mesh type) (re-operation) (one of them mortality due to gastric fistula causing sepsis)
- One patient with hydro-pneumothorax (chest tube insertion)
- One patient with pneumonia (mortality due to sepsis)
- One patient with cardiac arrhythmia (pace maker insertion)
- One patient with myocardial infarction(coronary stent)

More favorable post-operative patient satisfaction was found among type III GPEH patients, with less incidence of post-operative dysphagia (*p* = 0.002), and lower rate of 30-day re-admission (*p* = 0.005), The previous data are presented in Table 3.

**Table 3:** Follow-up data

Variable	All patients	Type III hernia	Type IV hernia	*p* value
Satisfaction				0.004*
Very satisfied	51 (39.2)	40 (44.4)	11 (27.5)	
Satisfied	48 (36.9)	36 (40)	12 (30)	
Neutral	23 (17.7)	12 (13.3)	11 (27.5)	
Dissatisfied	8 (6.2)	2 (2.2)	6 (15)	
Very dissatisfied	0(0)	0(0)	0(0)	
Re-operation	6 (4.6)	–	6 (15)	< 0.001*
Clavien grade ≥ 3	13 (10)	3 (3.3)	10 (25)	< 0.001*
Post-operative morbidity no. (%)	24 (18.5)	7 (7.8)	17 (42.5)	< 0.001*
Post-operative mortality	2 (1.5)	0 (0)	2 (5)	0.033
Post-op dysphagia				
No	107 (82.3)	81 (90)	26 (65)	0.002*
Temporary	17 (13.1)	6 (6.7)	11 (27.5)	
Persistent	6 (4.6)	3 (3.3)	3 (7.5)	
30-day re-admission	12 (9.2)	4 (4.4)	8 (20)	0.005*
Recurrence	27 (20.7)	12 (13.3)	15 (37.5)	0.002
Redo surgery for recurrence	12 (9.2)	7 (7.8)	5 (12.5)	0.391*
Hospital stay (days) (mean, range)	3 (2–21)	2.8 (2–5)	5.8 (2–21)	0.001**

*Chi-square test

**Mann–Whitney test

As shown in Table 4, univariate analysis revealed that old age, larger crural defect size, higher ASA class, weak crural musculature, type IV GPEH, and mesh cruroplasty were significant risk factors for peri-operative morbidity. Only type IV GPEH maintained its significance in the multivariate analysis (*p* = 0.001).

**Table 4:** Risk factors of peri-operative morbidity

Variable mean (SD)	Morbidity		*p* value	HR (95%CI)	*p*
	Yes (24)	No (106)			
Age	61.3 (15.2)	54.6 (12.6)	0.025	0.8 (0.4–1)	0.99
BMI	26 (4.2)	26.1 (3.6)	0.929		
Hemoglobin	11.1 (1.9)	10.8 (1.7)	0.467		
Albumin	4 (0.4)	4 (0.2)	0.88		
Crura defect size	7.5 (1.1)	6.7 (0.96)	< 0.001	1.5 (0.7–2.1)	0.913
Sex			0.867		
Male	12 (50)	55 (51.9)			
Female	12 (50)	51 (48.1)			
Smoking	8 (33.3)	28 (26.4)	0.494		
ASA class			0.018	0.9 (0.7–1.3)	0.106
I	8 (33.3)	59 (55.7)			
II	8 (33.3)	35 (33)			
III	8 (33.3)	12 (11.3)			
Chest disease	7 (29.2)	29 (27.4)			
			0.858		
Crura condition			0.035	1.1 (0.6–1.3)	0.544
Weak	9 (37.5)	19 (18)			
Good	15 (62.5)	87 (82)			
Wrap type			0.759		
Nissen	20 (83.3)	81 (76.4)			
Dor	1 (4.2)	7 (6.6)			
Toupet	3 (12.5)	18 (17)			
Hernia type			< 0.001	1.6 (1.2–1.9)	0.001
III	7 (29.2)	83 (78.3)			
IV	17 (70.8)	23 (21.7)			
Gastropexy	13 (54.2)	41 (38.7)	0.164		
Dysphagia	12 (50)	37 (35)	0.168		
Barrett’s esophagus	1 (4.2)	3 (2.8)	0.732		
Peptic stricture	0 (0)	6 (5.6)	0.233		
Relaxing incision	7 (29.2)	25 (23.6)	0.567		
Repair method			0.036	0.5 (0.2–0.8)	0.921
Primary repair	18 (75)	96 (90.6)			
Mesh use	6 (25)	10 (9.4)			
Surgeon experience	7 (29.2)	34 (32.1)	0.782		
< 10 years	17 (70.8)	72 (67.9)			
> 10 years					

### Risk factors of recurrence

Hernia recurrence was defined as > 2 cm vertical extension of the gastric mucosa above the level of the diaphragm (Reviewer #2).

With a mean follow-up of 65 months (range 48–150 months), Recurrence of HH was encountered in 27 (20.7%) patients, with a significant increase in type IV GPEH patients (*p* = 0.002).

Twelve of them required a redo surgery, 8 patients with asymptomatic recurrence, while the remaining patients were conservatively managed with medications. The previous data are summarized in Table 3.

On the assessment of predictors for post-operative recurrence (Table 5), low pre-operative albumin level, larger crural defect, weak diaphragmatic musculature, type IV GPEH, and low surgeon experience were the significant risk factors in the univariate analysis. All previous variables maintained their significance in the multivariate analysis, apart from weak diaphragmatic musculature.

**Table 5:** Risk factors of recurrence

Variable Mean (SD)	Recurrence		*p* value	HR (95% CI)	*p*
	No (103)	Yes (27)			
Age	55.1 (12.6)	58.6 (15.8)	0.219		
BMI	26.1 (3.7)	25.9 (3.8)	0.805		
Hemoglobin	10.9 (1.7)	10.8 (1.8)	0.787		
Albumin level	4.1 (0.2)	3.9 (0.3)	0.002	1.9 (1.1–2.3)	0.033
Crura defect size	6.6 (0.9)	7.7 (1.1)	< 0.001	1.8 (1.3–2.1)	0.039
Sex			0.892		
Male	54 (52.4)	13 (48.2)			
Female	49 (47.6)	14 (51.8)			
Smoking	29 (28.2)	7 (25.9)	0.818		
ASA class					
I	56 (54.4)	11 (40.7)	0.199		
II	34 (33)	9 (33.3)			
III	13 (12.6)	7 (26)			
Chest disease	27 (26.2)	9 (33.3)	0.462		
Crura condition					
Weak	17 (16.5)	11 (40.7)	0.006	1.2 (0.5–1.5)	0.29
Good	86 (83.5)	16 (59.3)			
Wrap type					
Nissen	79 (76.7)	22 (81.5)	0.709		
Dor	6 (5.8)	2 (7.4)			
Toupet	18 (17.5)	3 (11.1)			
Hernia type					
III	78 (75.7)	12 (44.6)	0.002	1.4 (1–1.8)	0.01
IV	25 (24.3)	15 (55.5)			
Additional gastropexy	41 (39.8)	13 (48.1)	0.434		
Post-operative dysphagia	39 (37.9)	10 (37)	0.937		
Barrett’s esophagus	2 (2)	2 (7.4)	0.143		
Peptic stricture	4 (3.9)	2 (7.4)	0.437		
Relaxing incision	29 (28.2)	3 (11.1)	0.1		
Repair method					
Primary repair	91 (88.3)	23 (85.2)	0.656		
Mesh use	12 (11.7)	4 (14.8)			
Operator experience			< 0.001	2 (1.2–2.3)	0.026
< 10 years	23 (22.3)	18 (66.7)			
> 10 years	80 (77.7)	9 (33.3)			

### Quality of life assessment

Pre-operative GIQLI scores were comparable between the two GPEH groups, and both groups experienced an improvement in their quality of life after laparoscopic repair and at 1-, 3-, 4-year follow-up, irrespective of the GPEH type (Table 6).

**Table 6:** Post-operative and follow-up changes in GIQL index score

Variable Mean, SD	Hernia type			*p* value
	All patients	Type III	Type IV	
Pre-GIQL score *n* = (100)	76.5 (8.1)	76.2 (8.3) *n* = (70)	76.8 (7.8) *n* = (30)	0.791
Post-GIQL *n* = (100)	108.9 (9.02)	109.97 (8.6) *n* = (70)	106.3 (9.6) *n* = (30)	0.063
1-year GIQL *n* = (100)	109.4 (7.6)	109.6 (7.6) *n* = (70)	108.8 (7.5) *n* = (30)	0.643
3-year GIQL *n* = (90)	110.7 (7.6)	110.9 (7.1) *n* = (63)	110.2 (8.7) *n* = (27)	0.687
4-year GIQL *n* = (82)	111.1 (6.3)	111.6 (5.7) *n* = (59)	110.3 (7.3) *n* = (23)	0.438

However, patients with mesh cruroplasty or post-operative recurrence showed a significant decline in the same score compared to patients without the previous criteria, Tables 7 and 8.

**Table 7:** GIQL index score changes between recurrent and non-recurrent group

Variable	Recurrence		*p* value
	No	Yes	
Pre-GIQL *n* = (100)	76.4 (8.1) *n* = (78)	76.7 (8.5) *n* = (22)	0.904
4-year GIQL *n* = (82)	112.5 (5.9) *n* = (60)	106.4 (5.3) *n* = (22)	0.001

**Table 8:** GIQL index score between primary repair and mesh repair group

Variable	Repair		*p* value
	Primary	Mesh	
Pre-GIQL *n* = (100)	76.8 (7.6) *n* = (87)	73.7 (12.1) *n* = (13)	0.379
4-year GIQL *n* = (82)	111.7 (6) *n* = (69)	105.2 (6) *n* = (13)	0.014

## Discussion

The current investigation included 130 patients with type III (*n* = 90) and type IV (*n* = 40) GPEH with the primary outcome to compare between them regarding peri-operative morbidity, recurrence, and quality of life.

No standard definition of GPEH has been advocated; in our study, we included patients with > 30% prolapsed stomach with or without other organs in the pre-operative imaging [14]. Others adopted an intra-operative definition of GPEH with a hiatal defect wider than 8 cm [14, 21, 22] or more than 50% of the stomach prolapsed in the thoracic cavity [4, 23].

On comparing type III and type IV GPEHs, we found that type IV GPEH patients were older, more fragile, and scored worse on the ASA physical classification score, aside from having a more challenging surgical technique (wider crura, weaker muscles, increased need for release incisions, mesh cruroplasty, and gastropexy procedures).

The previously mentioned factors have contributed to the significantly higher rate of post-operative morbidity and complications (25% vs. 3.3% had > grade 3 Clavien–Dindo score in patients with types IV and III GPEH, receptively—*p* < 0.001).

Moreover, the re-operation rate was significantly higher in type IV patients (15% vs 0% in type III patients—*p* < 0.001). Also, hospital re-admission rate was significantly higher with type IV GPEH patients. The only two patients who died in the peri-operative period had type IV GPEH.

In the study conducted by Rodríguez-Luna et al., which included 91 type III and 12 type IV GPEH patients, The authors reported that type IV GPEH patients had significantly older age (*p* = 0.039), worse co-morbidity index (*p* = 0.016), and were associated with more post-operative complication rates (*p* < 0.001). They also reported one case of post-operative mortality of type IV hernia. Nonetheless, they reported no significant difference regarding need for mesh cruroplasty, hospital re-admission, or reparation rates between both groups (*p* = 0.731, 0.888, and 0.235, respectively) [24].

Dara et al. also reported no significant difference between the two groups regarding post-operative complications (*p* = 0.323), but this study included only five patients with type IV GPEHs, which explains the absence of statistical significance [25].

The reported data comparing type III and type IV GPEHs are scarce due to the low incidence of their presentation. However, the increased rate of post-operative complications led to conflict between authors regarding type IV GPEH management. Some authors have advocated the watchful waiting strategy for those patients owing to the lower rates of acute presentations when compared to higher rates of post-operative complications [26–28].

### Hernia recurrence and its predictors

In this study, we had 27 patients of hernia recurrence (20.7%) among our patients a minimum follow-up of 48 months, which were significantly higher in type IV GPEH patients (37.5%) compared to type III GPEH patients (13.3%), while redo surgery was needed in 9.2% of our patients (12/27—44% of recurrence cases). Our total incidence rate lies within the reported range in the literature that was previously mentioned in the “Introduction” section.

Rodríguez-Luna et al. reported a 15.5% recurrence rate among their series, which included 103 patients with no significant difference between type III and type IV GPEH patients, and their re-operation rate was 2.9% [24]. In the study conducted by Lidor et al., which included 70 patients with type III GPEHs, they reported a recurrence rate of 27% (19 cases), and 4 cases (5.7%) underwent revisional surgeries [29].

Another study conducted by Targarona et al. included 55 patients with a median follow-up period of 108 months. They reported a relatively higher rate of radiological recurrence (49%). However, the authors declared that all recurrence cases had small hiatus hernias (< 3 cm), and they had a significant reduction in their quality of life compared to non-recurrence patients. None of the recurrence cases required re-operation. In the same study, the authors reported a 21% recurrence rate with a median follow-up period of 26 months, which highlights that recurrence is also time dependent [30].

We noticed a wide range (12–65%) regarding the recurrence rate among different authors, which could differ according to the number of included cases, the duration of follow-up, surgical expertise, and intra-operative technical issues. Re-operation rate also varies between studies as it ranges between 7 and 11% [7, 30–35].

Regarding risk factors of hernia recurrence, we found low serum albumin, large crural defects, weak crural musculature, type IV GPEH, and low operator experience level to be significant factors on the univariate analysis; while, most of these factors maintained their statistical significance on the multivariate analysis. To the best of our knowledge, there is paucity of studies evaluating risk factors for recurrence in patients with GPEH. Most studies only discussed recurrence after hiatus hernia repair [36, 37], not focusing on GPEH, as we did.

Previous studies have reported that adequate esophageal mobilization, cruroplasty, mesh reinforcement, fundoplication, and gastropexy were protective against post-operative recurrence [38, 39]. In our study, we used a tailored surgical procedure without randomization; the need for mesh cruroplasty, release incision, and gastropexy were performed according to our assessment of intra-operative tension across the hiatus.

Lidor et al. concluded that larger paraesophageal hernias have a greater tendency to recur. However, they could not identify significant risk factors for recurrence (all *p* values > 0.05). It is worthy to mention that all of their cases received biological mesh cruroplasty, and they reported recurrence rates of 21% and 49% at 26 and 108 months, respectively [29].

### Mesh cruroplasty

Technical pearls to prevent hernia recurrence were investigated by many authors over a long period of laparoscopic surgery evolution. Mesh cruroplasty was advocated as a critical step against hernia recurrence. Our experience included mainly the use of composite mesh in the presence of intra-operative indications as described before. Initially, we had two complicated cases of mesh erosion with polypropylene mesh (Reviewer #2), so we decided to quit using this type of mesh. Additionally, we did not use biological mesh due to limited availability, very high cost and unfavorable results on long-term basis.

We found that mesh cruroplasty did not significantly affect hernia recurrence. However, surprisingly, it was found significant as a risk factor for peri-operative morbidity on univariate analysis (*p* = 0.036). Also, we found that, patients with mesh cruroplasty showed a significant decline in GIQL score compared to patients without mesh.

Actually, the ideal mesh cruroplasty is still an unanswered question among authors regarding its type, indication, site of insertion, and interpretation of results. Some randomized controlled trials reported that mesh cruroplasty is associated with significantly lower recurrence rates [9, 22, 23, 40, 41]. On the other hand, Dallemagne et al. reported no significant difference between both groups in terms of hernia recurrence after a mean follow-up of 99 months [42].

Moreover, mesh complications show a wide range of variation between different studies. Huddy et al., in their meta-analysis reported 0% of mesh-related complication [43]. In another study conducted by Targarona et al., the authors performed mesh cruroplasty in 9 out of 77 of their cases. Five of their cases developed severe dysphagia, and three of them required re-operation [30].

### Diaphragmatic relaxing incision

Another technical pearl to overcome hiatal tension is the creation of a diaphragmatic release incision. We selectively utilized this technique in 32 (24.6%) patients of our series which was significantly needed for type IV hernia patients (18 of 40) as compared to type III patients (14 out of 90).Noteworthy, the relaxing incision site was reinforced with composite mesh only with weak crural muscles. That maneuver did not result in significant increase in post-operative morbidity.

One study conducted by Crespin et al. compared the outcomes of suture cruroplasty, biological mesh cruroplasty, and relaxing incision with reinforcement with biologic mesh. They concluded that at a median follow-up of 9 months (6–83 months), there was no significant difference between the three groups of patients regarding hernia recurrence (*p* = 0.428) [44].

However, re-operation was needed in four patients in the previous study; two cases for recurrent hiatal hernia after suture cruroplasty and mesh cruroplasty, and two cases developed diaphragmatic hernia at site of reinforced left crural relaxing incision (*n* = 2/16), which makes the role of biologic mesh questionable [44].

### Short esophagus

Short esophagus is a good topic for argument among upper gastrointestinal surgeons. Does it really exist? What should you do when you face a case with a short esophagus? In our experience, we claim that, even when apparent short esophagus (*n* = 24) is present in the beginning of hiatal dissection, this should fade away with extensive mediastinal dissection. Actually, that was all we needed, and no lengthening procedures were warranted in our series.

This concept came in line with other studies questioning the concept of short esophagus [11, 12]. Other authors declared that it might be a significant factor for failed repair in revisional surgeries. According to the study of Horgan et al. which included 31 redo patients, they identified short esophagus in only one patient (3%). However, extensive dissection achieved the optimal esophageal length in other patients [45].

In the study carried out by Nason et al., they reported their experience in patients with giant paraesophageal hernias, and they found no significant difference in radiological recurrence between Collis gastroplasty (*n* = 454) and traditional fundoplication (*n* = 341) [46].

### Post-operative quality of life

We believe that the patient quality of life is the essence of hiatal hernia investigation. Our patients declared marked improvement in their GIQL index when questioned at 1, 3, and 4 years after their procedure compared to the corresponding baseline values.

The vast majority of them found it was a wise decision to have their hernia surgically repaired. We also noted a marked impairment in quality of life in recurrence patients compared to non-recurrence ones.

Other studies showed a significant improvement in quality-of-life scores after surgery [30–32]. Additionally, Furtado et al. reported that post-operative recurrence was associated with a marked decline in GIQLI score [32]. The previous results coincide with our results.

### Study limitations

Our study handled a rare surgical point of view with a respectable patient number. However, they were collected from one single center, thus reflecting the opinion of one surgical team. The upcoming studies should include patients from different centers to express different opinions on the management of such a challenging surgical entity. Also, we recommend to develop standards for laparoscopic repair of GPEHs, the following should be considered:

A step towards standardization:

Standard definition of GPEH

Standard surgical procedure for

1. Measurement of hiatal defect and radial tension,
2. Indication for esophageal lengthening procedure,
3. Indication for relaxing incision,
4. Indication for mesh cruroplasty,
5. Ideal criteria for the hiatal mesh

## Conclusion

The management of GPEHs is challenging for gastrointestinal surgeons. Type IV GPEH has a higher peri-operative morbidity and recurrence rate, so a more tailored surgical procedure (Reviewer #2) with high surgeon experience in a high-volume center were needed. Handling modifiable risk factors is essential to decrease recurrence rate like the correction of hypoalbuminemia and assigning the operation for high-volume surgeons.
